# Genetic and epigenetic regulation of Treg cell fitness by autism-related chromatin remodeler CHD8

**DOI:** 10.1186/s11658-025-00711-z

**Published:** 2025-03-28

**Authors:** Jun-Qi Yang, Chen Wang, Ramesh C. Nayak, Manohar Kolla, Mingjun Cai, Mario Pujato, Yi Zheng, Q. Richard Lu, Fukun Guo

**Affiliations:** 1 Division of Experimental Hematology and Cancer Biology, Children’s Hospital Medical Center, Department of Pediatrics, University of Cincinnati College of Medicine, 3333 Burnet Avenue, Cincinnati, OH 45229 USA; 2 Life Sciences Computational Services LLC, Huntingdon Valley, PA 19006 USA

**Keywords:** CHD8, Treg, Treg plasticity, Chromatin remodeling, Gene expression

## Abstract

**Background:**

Chromatin remodeler chromodomain helicase DNA-binding protein 8 (CHD8) defines a subtype of autism that is associated with immune disorders. It remains unknown whether CHD8 plays a cell-intrinsic role in immune cells such as regulatory T cells (Tregs) that maintain immune tolerance through suppressing CD4^+^ and CD8^+^ effector T cells.

**Methods:**

Treg-specific conditional CHD8-deficient mice were generated by crossing Chd8^Flox/Flox^ mice with Foxp3^YFP−cre^ transgenic mice. Effects of CHD8 deficiency were investigated using hematoxylin and eosin (H&E) staining, flow cytometry, and multi-omics, including RNA-sequencing (RNA-seq), assay for transposase-accessible chromatin sequencing (ATAC-seq), and chromatin immunoprecipitation sequencing (CHIP-seq).

**Results:**

We found that Treg-specific CHD8 deletion led to early, fatal inflammation owing to increased CD4^+^ and CD8^+^ effector T cells. CHD8 deletion did not alter Treg homeostasis but increased their functional plasticity with elevated expression of effector T cell cytokines. CHIP-seq of Tregs uncovered that CHD8 binding genes were enriched in phosphatidylinositol-3 kinase (PI3K)–protein kinase B (Akt)–mammalian target of rapamycin (mTOR) signaling and several other pathways. RNA-seq and ATAC-seq revealed that CHD8 deletion upregulated a number of pathways, notably mammalian target of rapamycin complex 1 (mTORC1) signaling and its mediated glycolysis that have been reported to promote Treg plasticity. Integrating RNA-seq data with CHIP-seq and ATAC-seq data identified a number of CHD8 target genes whose expression depends on CHD8 direct binding-mediated chromatin remodeling.

**Conclusions:**

Our findings suggest that CHD8 plays an important role in maintaining Treg fitness through genetic and epigenetic mechanisms to control autoimmunity, which may have important implications in immune changes in autism.

**Supplementary Information:**

The online version contains supplementary material available at 10.1186/s11658-025-00711-z.

## Introduction

CD4^+^ regulatory T cells (Tregs) are a specialized subset of T cells that suppress the activation and proliferation of other immune cells, particularly conventional/effector T cells [1]. Tregs play a crucial role in maintaining immune tolerance. They are important in preventing autoimmune diseases, allergies, and other immune-related disorders [2, 3]. Tregs express high levels of forkhead box P3 (Foxp3), a transcription factor that is unique to Tregs and required for Treg development and suppressive function [4]. Recent studies indicate that Tregs possess functional plasticity, manifested by being skewed toward effector-like Tregs that express effector T cell cytokines [4, 5].

Chromodomain helicase DNA-binding (CHD) proteins are crucial regulators of chromatin remodeling, gene transcription, and expression. As ATP-dependent chromatin modifiers, CHD proteins control the access of transcription factors and RNA polymerases to DNA by either “opening” or “closing” the structure of chromatin, which serve as gatekeepers of genomic access and deposit histone variants required for gene regulation [6]. CHD proteins are indispensable for developmental processes and are well-known to regulate autism pathogenesis [7, 8]. CHD8 of the CHD proteins is involved in many important signaling pathways, such as p53 and Wnt-β-catenin pathways [9, 10]. It is one of the most frequently mutated genes involved in the autism spectrum disorder [7, 11, 12]. Patients with CHD8 mutations often present with a distinctive phenotype, which includes not only the typical features of autism, such as social communication deficits and repetitive behaviors, but also macrocephaly, craniofacial abnormalities, intellectual disability, and overgrowth. Recent studies have demonstrated that CHD8 haploinsufficiency leads to disrupted brain development, particularly in the areas responsible for social and cognitive functions. These findings underscore the importance of CHD8 in neurogenesis and brain development, offering insights into the molecular mechanisms underlying CHD8-mediated autism [8, 13].

We have reported that CHD8 is essential for regulating hematopoiesis [9], similar to several other CHD proteins, including CHD3, CHD4, and CHD7. Loss of CHD8 impairs hematopoietic stem and progenitor cell (HSPC) survival and hematopoiesis [9, 14]. CHD8 deficiency leads to defective erythroblast cytokinesis and erythroid differentiation [15]. To date, the cell-intrinsic role of CHD8 in immune cells remains unknown. Given that autism is often accompanied by inflammatory disorders, including autoimmunity [16–19], and that an imbalance between T helper 17 cells (Th17) and Tregs may play a vital role in the progression of autism [20], it is conceivable that CHD8 impacts the immune system.

In this study, we aimed to investigate whether CHD8 in Tregs affected Treg behaviors to implicate its role in immune changes in autism. By characterizing Treg-specific CHD8 knockout (KO) mouse models, we found that loss of CHD8 dampened Treg fitness, which was associated with genetic and epigenetic alterations, leading to early, fatal, effector T cell-mediated inflammation, offering an explanation for the immune disorders in autism.

## Materials and methods

### Mouse model

Chd8^Flox/Flox^ mice were used, as reported previously [21]. To delete CHD8 in vivo in Tregs, Chd8^Flox/Flox^ mice were mated with Foxp3^YFP−Cre^ mutant mice, which express a knocked-in yellow fluorescent protein (YFP)/iCre-recombinase fusion protein from the Foxp3 locus without disrupting expression of the endogenous Foxp3 gene (strain no. 016959, Jackson Laboratory, Bar Harbor, ME, USA). Mouse genotyping was performed by PCR with primers for wild type (WT), floxed (Flox), and KO allele of Chd8 or Foxp3-Cre. In each experiment, 5–7 week-old and sex-matched Chd8^−/−^ and their WT littermates were used.

### Cell preparation and flow cytometry

Splenocytes were prepared freshly from Chd8^−/−^ mice and their WT littermates. Tregs were isolated from spleen with a CD4^+^CD25^+^ Regulatory T Cell Isolation kit by AutoMACS Pro (Cat no. 130–091-041, Miltenyi Biotec, Auburn, CA, USA). The purity of isolated Tregs was checked by fluorescence-activated cell sorting (FACS). Where indicated, splenocytes were stimulated with phorbol 12-myristate 13-acetate (PMA) (25 ng/ml) plus ionomycin (500 ng/ml) (Sigma, St Louis, MO, USA) for 5 h with GolgiStop (BD Bioscience, San Jose, CA, USA) in the last 2 h. Cells were then processed for surface and intracellular FACS staining, and cell proliferation was evaluated by in vivo bromodeoxyuridine (BrdU) incorporation assay, as previously reported [22].

### Real-time quantitative reverse transcription PCR (qPCR)

Total RNA was extracted from splenic Tregs with the RNeasy Mini kit (Qiagen, Valencia, CA, USA), and cDNA was prepared and qPCR was performed with SYBR Green qPCR or TaqMan Gene Expression Master Mix (Life Technologies, Carlsbad, CA, USA) on a StepOnePlus Real-Time PCR System (Thermo Fisher Scientific), as previously reported [22].

### Histology

The organs from mice were fixed freshly with 4% paraformaldehyde in phosphate buffer saline (PBS) and processed for histological examination with hematoxylin and eosin (H&E) staining.

### RNA sequencing (RNA-Seq)

Splenic CD4^+^ T cells were isolated from WT and Chd8^−/−^ mice by AutoMACS Pro. YFP^+^ cells were then sorted by FACS and analyzed for gene expression profiles by RNA-Seq. The next generation sequencing technology (10X Genomics Next GEM 3 version 3.1 assay) was performed by the Single Cell Genomics Core, Cincinnati Children’s Hospital Medical Center. RNA-seq libraries were prepared using Illumina RNA-Seq Preparation Kit and sequenced using a Novaseq 6000 sequencer at the DNA core of Cincinnati Children’s Hospital Medical Center.

### Chromatin immunoprecipitation sequencing (CHIP-seq)

Splenic CD4^+^CD25^+^ Tregs were isolated from C57B6/J mice by AutoMACS Pro. Half million Tregs were used for ChIP-seq that was performed according to a previously described protocol with slight modifications [23, 24]. In brief, Tregs were crosslinked with 1% formaldehyde at room temperature for 15 min with rotation and then quenched with 125 mM glycine. Isolation of nuclei, extraction of chromatin, and shearing with sonication were carried out as previously described [25] using the Magna ChIP A/G kit (Millipore Sigma, catalog no. 17–10,085). Chromatin was immunoprecipitated by incubating soluble chromatin with 1.5 μg of CHD8 antibody (Bethyl Laboratories, no. A301–224) or 1.5 μg of immunoglobulin G (IgG) antibody (Abcam, ab37355) at 4 °C overnight. Magna ChIP® Protein A + G Magnetic Beads (Millipore Sigma, catalog no. 16–663) were added next day and incubated for 2 h. CHD8-bound beads were washed in low salt wash buffer, high salt wash buffer, LiCl wash buffer, and Tris-EDTA (TE) buffer subsequentially. Harvested chromatin was then eluted from the beads, crosslinks were reversed, and DNA was purified as previously described [25], using the MinElute PCR Purification Kit (QIAGEN no. 28,204). Libraries were prepared with the NEBNext® Ultra™ II DNA Library Prep Kit for Illumina (no. E7645). Barcoded libraries were sequenced using a Novaseq 6000 sequencer at the DNA core of Cincinnati Children’s Hospital Medical Center.

### Assay for transposase-accessible chromatin sequencing (ATAC-seq)

ATAC-seq was carried out as described by Buenrostro et al. [26]. Briefly, ~50,000 sorted WT and Chd8^−/−^ CD4^+^YFP^+^ Tregs were centrifuged at 500 × *g* for 20 min at 4 °C. Cells were lysed in ice cold lysis buffers (10 mM Tris-HCl pH 7.4, 10 mM NaCl, 3 mM MgCl2, and 0.1% NP-40) and centrifuged at 500 × *g* at 4 °C for isolation of nuclei. Transposase reaction was performed by incubating the isolated nuclei with Tn5 transposase from the Nextera DNA Library Preparation Kit (Illumina) for 30 min at 37 °C. Transposed DNA was purified using QIAGEN mini elute DNA purification kit per manufacturer’s instructions. PCR amplification and barcoding were done using forward (i5 Forward) and reverse (i7 Reverse) primers with specific index, NEB 2 × PCR Mix (New England Biolabs), and tagmented DNA. PCR conditions were the following: 72 °C for 5 min, 98 °C for 30 s, five cycles of 98 °C for 10 s, 63 °C for 30 s, and 72 °C for 1 min. After the first PCR, DNA size was selected with 0.5 × volume SPRI beads (Agencourt AMPure, Beckman Coulter) and cleaned up with 1.5 × SPRI beads. The second PCR (98 °C for 30 s, followed by five cycles of 98 °C for 10 s, 63 °C for 30 s, and 72 °C for 1 min) was performed with same forward and reverse primers. Libraries were purified with 1.5 × SPRI beads, and concentration and ATAC DNA profiles were analyzed with the Bioanalyzer DNA High Sensitivity Kit (Agilent). Libraries were sequenced on Novaseq 6000 (Illumina), with an average of 25 million paired-end reads per sample.

Primers used:

i5-1-Forward: AATGATACGGCGACCACCGAGATCTACACTAGATCGCTCGTCGGCAGCGTCAGATGTGTAT.

i7-1-Reverse: CAAGCAGAAGACGGCATACGAGATTCGCCTTAGTCTCGTGGGCTCGGAGATGTG.

i5-2-Forward: AATGATACGGCGACCACCGAGATCTACACCTCTCTATTCGTCGGCAGCGTCAGATGTGTAT.

i7-2-Reverse: CAAGCAGAAGACGGCATACGAGATCTAGTACGGTCTCGTGGGCTCGGAGATGTG.

i5-3-Forward: AATGATACGGCGACCACCGAGATCTACACTATCCTCTTCGTCGGCAGCGTCAGATGTGTAT.

i7-3-Reverse: CAAGCAGAAGACGGCATACGAGATTTCTGCCTGTCTCGTGGGCTCGGAGATGTG.

### Bioinformatic analysis

RNA-seq reads in FASTQ format were first subjected to quality control to assess the need for trimming of adapter sequences or bad quality segments. The programs used in these steps were FastQC version 0.11.7 [27], Trim Galore! version 0.4.2 [28], and cutadapt version 1.9.1 [29]. The trimmed reads were aligned to reference mouse genome version mm10 with the program STAR version 2.6.1e [30]. Aligned reads were stripped of duplicate reads with the program sambamba version 0.6.8 [31]. Gene-level expression was assessed by counting features for each gene, as defined in the NCBI’s RefSeq database [32]. Read counting was carried out with the program featureCounts version 1.6.2 from the Rsubread package [33]. Raw counts were normalized as transcripts per million (TPM). The list of differentially expressed genes and log2 fold changes were further used for gene set enrichment analysis (GSEA). GSEAPreranked tool within GSEA version 3.0.0 [34, 35] was applied with MH gene set from the Molecular Signatures Database (MSigDB) [36, 37]. Heatmap was generated using base graphics in R.

ATAC-seq and CHIP-seq raw reads in FASTQ files were assessed for quality control using FastQC version 0.11.9 [27]. Trimming of raw reads was performed using TrimGalore! version 0.6.7 [28] and cutadapt version 3.5 [29]. The trimmed reads were aligned to mouse reference genome mm39 with HISAT2 version 2.2.1-3n-0.0.2. [38]. Duplicate reads were removed using sambamba version 0.8.2 [31]. Peaks were called using Macs2 version 2.2.7.1 [39] and aggregated on each duplicate sample by combining overlapping peaks with bedtools version 1.16.1 [40]. A minimum of 50% overlap was considered. Peaks showing in only one replicate were removed. A final list of peaks was derived by taking the union of all aggregated peak lists. Reads under the final list of peaks were counted using featureCounts version 1.5.3 [41] on each binary alignment and map (BAM) file. Heatmaps and peak profiles were generated using deeptools version 3.5.5 (computeMatrix, plotHeatmap, and plotProfile) [42]. CHIP peaks were further overlapped with Chd8^−/−^ ATAC peaks. The overlapping Chd8^−/−^ ATAC peaks were split up into three classes: up, down, and same, according to their log2FC between WT and CHD8^−/−^ ATAC. ‘Regions’ 1.5 Kb around center of the described CHD8^−/−^ ATAC peaks were then employed to generate heatmaps and profiles using deeptools. These regions, defined by the overlapped Chd8^−/−^ ATAC peaks, were used to pair WT and CHD8^−/−^ regions and plotted side by side in heatmaps. In parallel, Chd8^−/−^ ATAC peaks non-overlapping with CHIP peaks were processed similarly to CHD8^−/−^ ATAC peaks overlapping with CHIP peaks. Two methods were used for gene set enrichments: (1) GSEA [34] when using a ranked list (the list of genes is ranked by the log2FC values) and (2) Enrichr [43–45]. Out of the complete Enrichr output, only the hallmark gene set was used, with the ten most enriched gene sets. Venn diagrams and bar graphs were generated using the R environment and the ggplot2 package [46].

### Statistical analysis

Two-tailed Student’s *t* or Mann–Whitney U test were used for experimental data analysis. *P* value of < 0.05 was considered significant.

## Results

### CHD8 deficiency causes effector T cell-mediated inflammatory disorders

To investigate the role of CHD8 in Tregs, we crossed Chd8^Flox/Flox^ mice [21] with Foxp3^YFP−cre^ transgenic mice, which express a knocked-in YFP/iCre-recombinase fusion protein under the control of X chromosome-linked Foxp3 gene, to generate Treg-specific Chd8^−/−^ mice (Chd8^Flox/Flox^Foxp3^YFP−Cre^ male mice or Chd8^Flox/Flox^Foxp3^YFP−Cre/YFP−Cre^ female mice) (Supplementary Fig. 1A). Splenic Tregs were purified from Chd8^−/−^ mice and WT mice (Chd8^+/+^Foxp3^YFP−Cre^ male mice or Chd8^+/+^Foxp3^YFP−Cre/YFP−Cre^ or Chd8^+/+^Foxp3^YFP−Cre/+^ female mice) by AutoMACS using a CD4^+^CD25^+^ regulatory T Cell Isolation kit. The purity of isolated Tregs was confirmed by FACS showing over 95% of CD4^+^CD25^+^ cells (Supplementary Fig. 1B). Tregs were genotyped by PCR for Chd8 and Cre. Chd8^−/−^ Tregs showed a strong KO allele with a faint Flox allele (Supplementary Fig. 1C). The faint flox allele was likely from the small proportion of non-Tregs. Through qRT-PCR analysis, we found that Chd8 expression was markedly reduced in Chd8^−/−^ Tregs (Supplementary Fig. 1D).

**Fig. 1:**
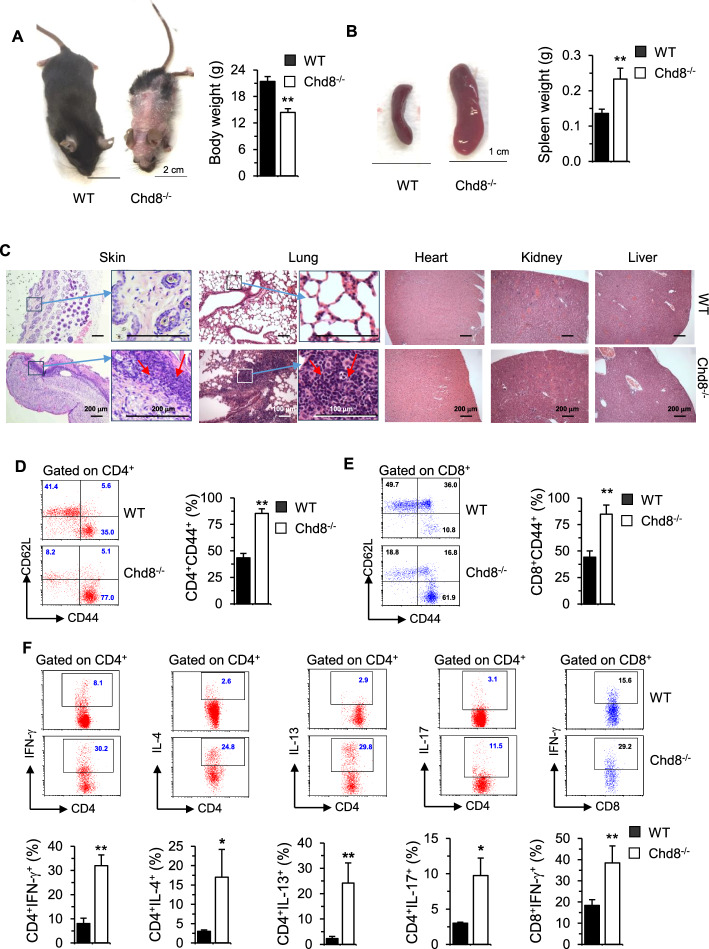
CHD8 deficiency causes effector T cell-mediated inflammatory disorders. **A**–**C** WT and Chd8^−/−^ mice were sacrificed at around 4–5 weeks old. Photos of mice **A**, spleens **B**, and their weights (right) are shown. Scale bars equal 1 cm or 2 cm, as indicated. WT: three males + two females; Chd8^−/−^: two males + two females. Mouse tissues, as indicated, were processed for H&E staining. Representative images are shown (**C**). The magnified views of skin and lung are also shown on the right of the original images, with red arrows pointing to the infiltrating leukocytes (scale bars equal 100 µm or 200 µm, as indicated). **D**–**E** Freshly isolated splenocytes were stained for CD4, CD8, CD62L, and CD44, followed by FACS analysis. Representative dot plots of CD62L and CD44 staining gated on CD4^+^ (**D**) or CD8^+^ (**E**) cells are shown (left). The percentages of CD44^+^ cells are shown in bar graphs (right). **F** Splenocytes were stimulated with PMA plus ionomycin for 5 h, with BD GolgiPlug™ added during the last 2 h. The cells were collected for surface staining of CD4 and CD8 and intracellular cytokine staining for IFN-γ, IL-4, IL-13, and/or IL-17, gated on CD4^+^ or CD8^+^cells, as indicated. Representative dot plots (upper) and percentages of cytokine positive CD4^+^ or CD8^+^ cells in bar graphs (lower) are shown. Results in bar graphs (**D**–**F**) represent mean plus standard deviation (SD) (WT: five males + five females; Chd8^−/−^: three males + five females). *P < 0.05, **P < 0.01 versus WT control group

Chd8^−/−^ mice were small, lacked mobility, lost hair, developed skin ulceration, and deceased in 5–7 weeks after birth (Fig. 1A, data not shown). The mice showed enlarged spleen (Fig. 1B) and massive leukocyte infiltration and/or distorted architecture in the skin and lung, with intact heart, kidney, and liver (Fig. 1C). CHD8 deficiency decreased the frequencies of CD4^+^ but not CD8^+^ T cells. However, the numbers of CD4^+^ T cells were not affected and the numbers of CD8^+^ T cells were increased, likely owing to increased total splenocytes (Supplementary Fig. 2). CHD8 depletion upregulated the proportions of CD4^+^CD44^+^ (Fig. 1D) and CD8^+^CD44^+^ (Fig. 1E) T cells.

To evaluate the potential effects of CHD8 deficiency on T cell cytokine secretion, spleen cells from both genotypes were briefly activated ex vivo with PMA plus ionomycin to induce cytokine production. GolgiStop was added in the last 2 h to block the intracellular protein transport processes required for the subsequent intracellular cytokine staining by FACS. We found that effector cytokines, namely interferon-gamma (IFN-γ), interleukin-4 (IL-4), IL-13, and/or IL-17 were significantly upregulated in CD4^+^ and CD8^+^ (Fig. 1F) T cells from Chd8^−/−^ mice. Given that IL-4 and IL-13 are known to promote allergic airway inflammation [22, 47], their increase may have contributed to the lung inflammation in CHD8^−/−^ mice. However, IL-4 and IL-13 could inhibit inflammatory response [48]. It is thus plausible that the increased IL-4 and IL-13 acted to resolve IFN-γ- and IL-17-induced inflammation but IFN-γ and IL-17 outperformed IL-4 and IL-13, resulting in net inflammation. Overall, these findings suggest that CHD8 deficiency induced effector T cell activation, which led to the observed inflammatory diseases.

### CHD8 deficiency does not affect Treg homeostasis

**Fig. 2:**
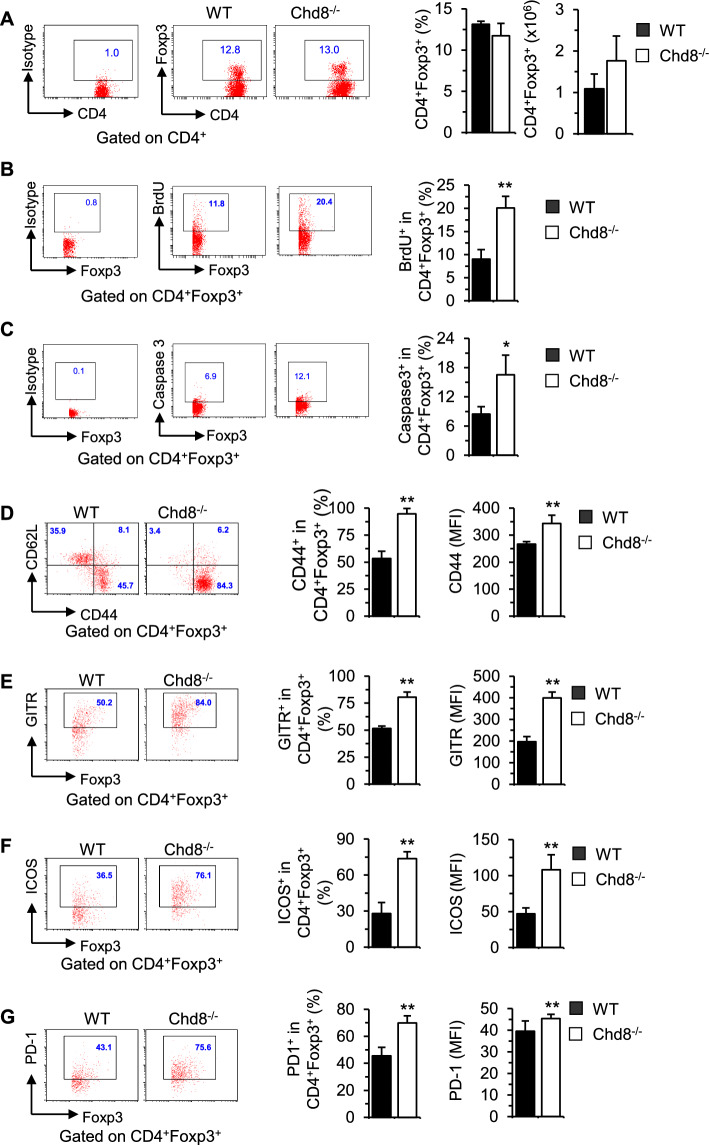
CHD8 deficiency does not affect Treg homeostasis but causes Treg activation. **A** Freshly isolated splenocytes were subjected to surface staining of CD4 and intracellular staining of Foxp3 (versus isotype control) and then analyzed by FACS. Representative dot plots of Foxp3 staining gated from CD4^+^ cells are shown. The graph panels show the proportions and absolute numbers of Tregs. **B** Treg proliferation. BrdU was injected into WT and Chd8^−/−^ mice 16 h before splenocytes were harvested for surface staining of CD4 and intracellular staining of Foxp3 and BrdU (versus isotype control). Representative dot plots of BrdU staining gated on CD4^+^Foxp3^+^ cells are shown. The percentages of CD4^+^Foxp3^+^ Tregs incorporated with BrdU are shown in a bar graph. **C** Treg apoptosis. Splenocytes were subjected to surface staining of CD4 and intracellular staining of Foxp3 and active caspase 3 (versus isotype control). Representative dot plots of active caspase3 staining gated on CD4^+^Foxp3^+^ cells are shown. The percentages of CD4^+^Foxp3^+^ Tregs expressing active caspase 3 are shown in a bar graph. **D** Splenocytes were subjected to surface staining of CD4, CD62L, and CD44 and intracellular staining of Foxp3. Representative dot plots of CD62L and CD44 staining gated on CD4^+^Foxp3^+^ cells are shown. The percentages and mean fluorescence intensity (MFI) of CD44^+^ cells are shown in bar graphs. **E–G** Splenocytes were subjected to surface staining of CD4 **E**–**G**, GITR **E**, ICOS **F**, and PD-1 **G**, and intracellular staining of Foxp3 **E**–**G**. Representative dot plots of GITR, ICOS, and PD-1 staining gated on CD4^+^Foxp3^+^ cells are shown. The percentages and MFI of the positively stained cells are shown in bar graphs. Results in bar graphs represent mean plus SD (WT: five males + five females; Chd8^−/−^: three males + three females). *P < 0.05, **P < 0.01 versus WT control group

Chd8^−/−^ mice did not show alterations in the frequencies and absolute numbers of Tregs (Fig. 2A). Thymic Treg output may affect peripheral Treg homeostasis. We thus examined thymocyte development in Chd8^−/−^ and WT mice. We found that Chd8^−/−^ mice had comparable CD4^−^CD8^−^, CD4^+^CD8^+^, and CD4^+^CD8^−^ thymocytes with a moderate decrease in CD4^−^CD8^+^ cells to WT mice (Supplementary Fig. 3A). Consistent with intact peripheral Treg homeostasis, the frequencies of thymic Tregs remained unchanged in Chd8^−/−^ mice (Supplementary Fig. 3B). Hence, it seems that CHD8 deficiency did not affect overt thymocyte/thymic Treg cell development. We next examined Chd8^−/−^ Treg proliferation and survival. We found that Chd8^−/−^ Tregs exhibited increased proliferation, as reflected by elevated BrdU incorporation (Fig. 2B) but were more susceptible to cell death, as evidenced by upregulated active caspase 3 (Fig. 2C). We reason that counterbalance between increased proliferation and decreased survival led to no changes in Treg homeostasis.

### CHD8-deficient Tregs are activated, plastic, and impaired in functional competitiveness with CHD8-proficient Tregs

**Fig. 3:**
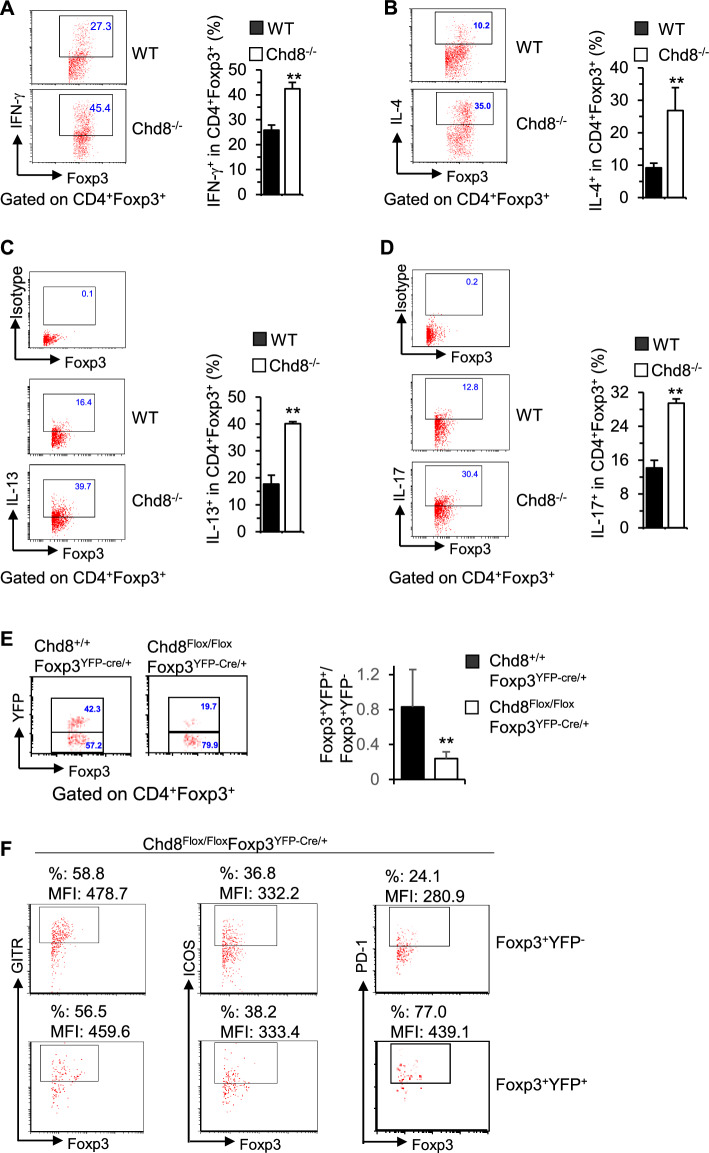
CHD8-deficient Tregs become plastic and are outcompeted by CHD8-proficient Tregs. **A**–**D** Freshly isolated splenocytes were stimulated with PMA plus ionomycin for 5 h with BD GolgiPlug™ added during the last 2 h. The cells were collected for surface staining of CD4 and intracellular staining of Foxp3, IFN-γ IL-4, IL-13, and IL-17. Percentages of IFN-γ^+^ (**A**), IL-4^+^ **B**, IL-13^+^ (**C**), and IL-17^+^ (**D**) cells gated on CD4^+^Foxp3^+^ cells are shown in representative dot plots and summarized in bar graphs. **E** Percentages of YFP^+^ and YFP^−^ cells gated on CD4^+^Foxp3^+^ cells are shown in representative dot plots from Chd8^+/+^Foxp3^YFP−cre/+^ and Chd8^Flox/Flox^Foxp3^YFP−cre/+^ mice. The ratios of YFP^+^/YFP^−^ cells are summarized in a bar graph. **F** Expression of the indicated Treg functional markers on Foxp3^+^YFP^−^ and Foxp3^+^YFP^+^ cells from Chd8^Flox/Flox^Foxp3^YFP−cre/+^ mice. Numbers indicate % and MFI. Data are representative of five mice. Results in bar graphs represent mean plus SD (WT: three males + three females; Chd8^−/−^: two males + three females). **P < 0.01 versus WT control group

CHD8 deficiency significantly upregulated Treg activation marker CD44 (Fig. 2D) and several functional markers, including glucocorticoid-induced TNFR-related protein (GITR) (Fig. 2E), inducible T cell costimulator (ICOS) (Fig. 2F), and programmed death 1 (PD-1) (Fig. 2G) on Tregs. These data suggest that deletion of CHD8 leads to spontaneous Treg activation. In addition, CHD8 deficiency upregulated effector T cell cytokines IFN-γ (Fig. 3A), IL-4 (Fig. 3B), IL-13 (Fig. 3C), and IL-17 (Fig. 3D) in Tregs. The results suggest that CHD8 deficiency converted Tregs to effector-like Tregs and thus CHD8^−/−^ Tregs became plastic.

We then examined female Chd8^Flox/Flox^Foxp3^YFP−Cre/+^ mice in comparison with female Chd8^+/+^Foxp3^YFP−Cre/+^ mice that were heterozygous for Foxp3^YFP−Cre^. As both CHD8-deficient Foxp3^+^YFP^+^ Tregs and CHD8-proficient Foxp3^+^YFP^−^ Tregs were present in female Chd8^Flox/Flox^Foxp3^YFP−Cre/+^ mice, the mice did not show signs of disease or weight loss. Owing to the X chromosome-linked nature of Foxp3 and random X chromosome inactivation by Foxp3^YFP−Cre^ knock-in transgene, female Foxp3^YFP−Cre/+^ mice are expected to maintain ~50% Foxp3^+^YFP^+^ Tregs and ~50% of Foxp3^+^YFP^−^ Tregs [4]. Indeed, female Chd8^+/+^Foxp3^YFP−Cre/+^ mice showed ~1:1 ratio of Foxp3^+^YFP^+^/Foxp3^+^YFP^−^ cells (Fig. 3E). Nonetheless, female Chd8^Flox/Flox^Foxp3^YFP−Cre/+^ mice had only ~0.25:1 ratio of Foxp3^+^YFP^+^/Foxp3^+^YFP^−^ (Fig. 3E). Thus, CHD8-deficient Foxp3^+^YFP^+^ Tregs were outcompeted by CHD8-proficient Foxp3^+^YFP^−^ Tregs in the same female Chd8^Flox/Flox^Foxp3^YFP−Cre/+^ mice.

Although Treg functional markers GITR, ICOS, and PD-1 were all increased on Chd8^−/−^ Tregs from Chd8^−/−^ mice bearing homozygous Foxp3^YFP−Cre^ (Fig. 2 E–G), only PD-1 was elevated on CHD8-deficient Foxp3^+^YFP^+^ Tregs when compared with CHD8-proficient Foxp3^+^YFP^−^ Tregs in the same mice bearing heterozygous Foxp3^YFP−Cre^ (Fig. 3F). Noting that PD-1 is a negative regulator of Treg function [49], our data suggest that loss of functional competence of CHD8-deficient Tregs is attributable to increased PD-1 expression. However, increased GITR and ICOS expression on Chd8^−/−^ Tregs from inflammatory Chd8^−/−^ mice may reflect a compensatory effect of dampened Treg function and the resultant inflammation.

Together, these findings suggest that CHD8 deficiency compromises Treg fitness, as reflected by increased plasticity that may lead to their conversion to CD4^+^ effector T cells and dampened function in suppressing CD4^+^ and CD8^+^ effector T cells.

### CHD8 deficiency alters gene expression in Tregs

**Fig. 4:**
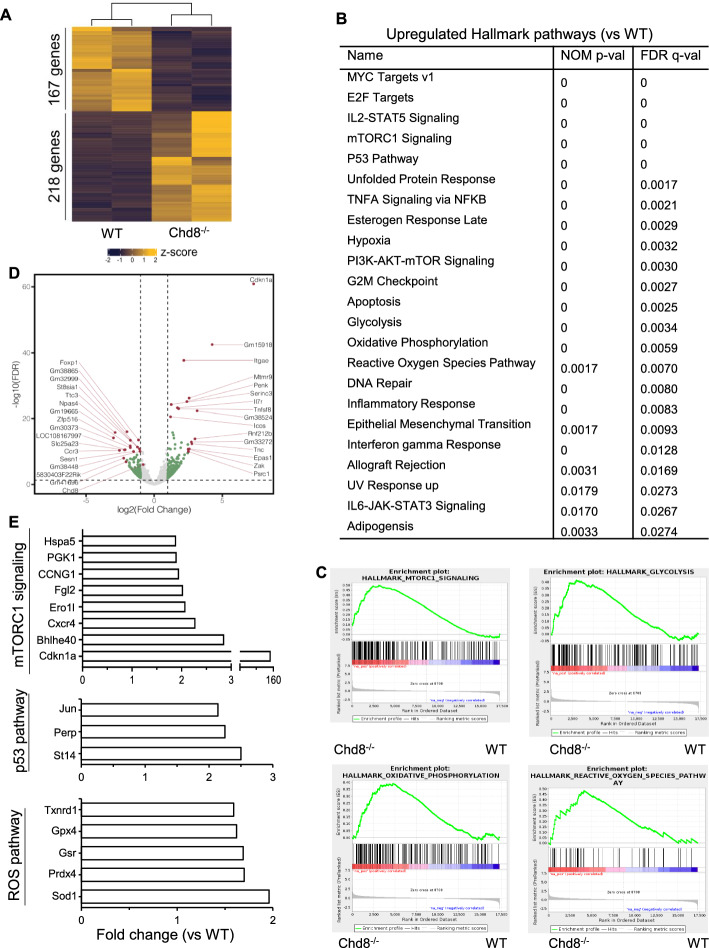
CHD8 deficiency alters global gene expression. Splenic CD4^+^ T cells were isolated from WT and Chd8^−/−^ mice (WT: one male + one female; Chd8^−/−^: one male + one female) by AutoMACS. YFP^+^ cells were then sorted by FACS and analyzed for gene expression profiles by RNA-Seq. **A** Heatmap of differentially expressed genes between WT and Chd8^−/−^ Tregs. **B** Representative upregulated hallmark pathways in Chd8^−/−^ Tregs. **C** Representative GSEA enrichment plots of upregulated pathways in Chd8^−/−^ Tregs. **D** Volcano plot of differentially expressed genes between WT and Chd8^−/−^ Tregs. Log2FC cutoff: 1.0; FDR < 0.05. **E** Fold changes (Chd8^−/−^ versus WT) of representative genes in mTORC1, p53, and ROS signaling pathways

Next, we explored potential mechanisms of CHD8-regulated Treg fitness. Sorted YFP^+^ Tregs were analyzed for gene expression profiles by RNA-Seq. Overall, 218 genes were upregulated, whereas 167 genes were downregulated in Chd8^−/−^ Tregs as compared with WT Tregs (Log2FC cutoff: 1.0) (Fig. 4A). Hallmark pathway analysis revealed that a number of pathways were upregulated, represented by mTORC1, p53, glycolysis, oxidative phosphorylation, reactive oxygen species (ROS), and inflammatory pathways such as IL-2-Stat5, which is important for IFN-γ expression, TNFA signaling via NFKB, inflammatory response, IFN-γ response, and IL-6-JAK-Stat3, which is essential for IL-17 expression (Fig. 4B and C). Of note, the most upregulated gene in Chd8^−/−^ cells was Cdkn1a (Fig. 4D), a target of various upregulated pathways such as mTORC1, p53, G2/M checkpoint, early region 2 binding factor (E2F), hypoxia, apoptosis, inflammatory response, IFN-γ response, and tumor necrosis factor alpha (TNFA) signaling via nuclear factor kappa-light-chain-enhancer of activated B cells (NFKB). The fold increase (Chd8^−/−^ versus WT) of representative genes in signaling pathways of mTORC1, p53, and ROS are shown (Fig. 4E), and upregulation of several key genes in these pathways was verified by qPCR (Supplementary Fig. 4).

### CHD8 regulates chromatin remodeling either dependent or independent of CHD8 direct binding

**Fig. 5:**
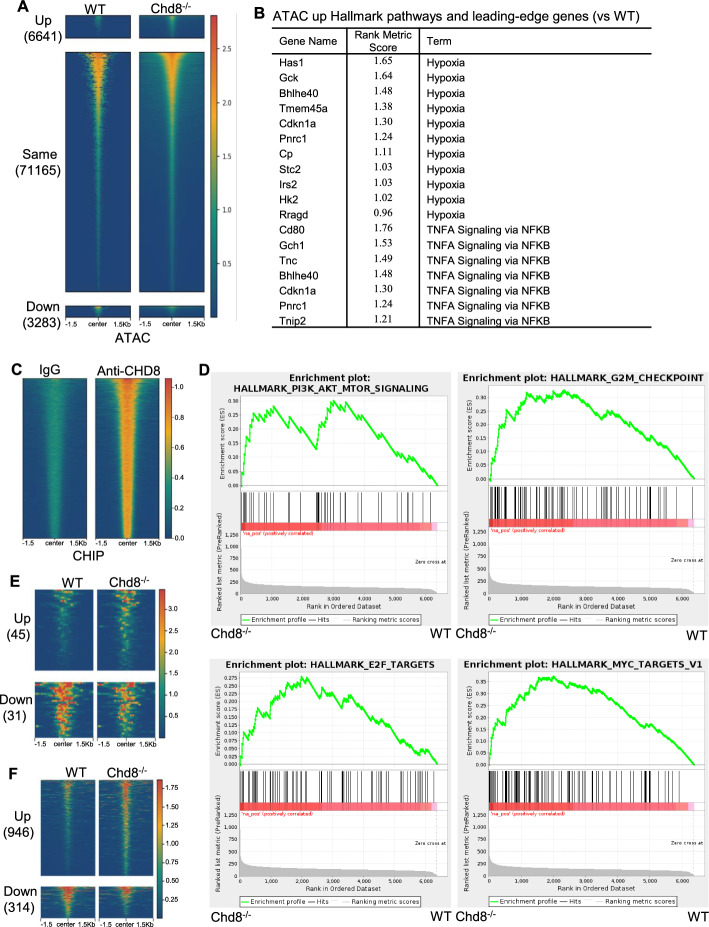
CHD8 deficiency leads to chromatin remodeling. Splenic CD4^+^ T cells were isolated from WT and Chd8^−/−^ mice by AutoMACS. YFP^+^ cells were then sorted by FACS and analyzed by ATAC-seq and CHIP-seq. **A** Heatmap of ATAC-seq peaks in WT and Chd8^−/−^ Tregs showing ± 1.5 kb around the ATAC-seq peak center. Results are from one WT male mouse, one Chd8^−/−^ male mouse, and one Chd8^−/−^ female mouse. Cutoff: Log2FC = 0.4. **B** Genes with altered ATAC-seq peaks in Chd8^−/−^ versus WT cells were analyzed for enrichment in hallmark pathways. Upregulated hallmark pathways and leading-edge genes are shown. Note: no downregulated hallmark pathways were found.** C** Heatmap of anti-CHD8 CHIP-seq peaks compared with IgG control in Tregs. Results are from one WT male mouse and one WT female mouse.** D** GSEA enrichment plot of representative pathway of CHD8 binding genes. **E** Heatmap of ATAC up and ATAC down peaks at CHD8 binding sites in Chd8^−/−^ Tregs.** F** Heatmap of ATAC up and ATAC down peaks at non-CHD8 binding sites in Chd8^−/−^ Tregs

CHD8 plays an important role in chromatin remodeling. We investigated whether CHD8 deficiency in Tregs affected chromatin remodeling, leading to altered chromatin accessibility. To this end, we performed an assay for transposase-accessible chromatin sequencing (ATAC-Seq) with sorted Tregs from WT and Chd8^−/−^ mice. Principal component analysis (PCA) revealed that chromatin accessibility in Chd8^−/−^ Tregs was clearly different from that in WT Tregs, and the Chd8^−/−^ biological duplicates exhibited similar chromatin accessibility (Supplementary Fig. 5A). Fragment size distribution analysis found that there was a high proportion of short fragments (< 100 base pairs (bp)) in each sample (Supplementary Fig. 5B), indicative of a successful ATAC-seq. A clear peak at ~200 bp was present in each sample (Supplementary Fig. 5B), suggesting that the ATAC-seq captured the expected pattern of nucleosome positioning. The fragment size distribution data also implicate that the library we prepared was of good quality. Heatmaps and peak profiles uncovered that CHD8 deficiency caused either increased (up) or decreased (down) chromatin accessibility (Fig. 5A; Supplementary Fig. 5C). Among all ATAC peaks, there were 6641 ATAC up and 3283 ATAC down peaks, whereas 71,165 peaks were not altered (same) by the loss of CHD8 (Fig. 5A), suggesting that CHD8 only regulates chromatin accessibility at a small proportion of genomic loci. Hallmark pathway analysis of genes with altered ATAC peaks upon CHD8 loss showed upregulation of hypoxia and TNFA signaling via NFKB, both of which contain mTORC1 target genes Cdkn1a and Bhlhe40 (Fig. 5B; Supplementary Fig. 5D).

CHD8 is a DNA binding protein. We thus carried out CHIP-seq to identify CHD8 binding sites in Tregs. PCA confirmed that CHD8 bound to DNA in Tregs and that the biological duplicates were reproducible (Supplementary Fig. 5E). The CHIP-seq found that most of the CHD8 binding peaks in Tregs were located within 5′ untranslated regions (UTR), transcription start sites (TSS), and exons (Fig. 5C; Supplementary Fig. 5F). Of note, TSS + 5′ UTR regions contained more than 80% of peaks, indicative of a strong enrichment around the promoter region. Hallmark pathway analysis of CHD8 binding genes showed enrichment in PI3K-Akt-mTOR signaling, G2M checkpoint, E2F targets, and Myc targets (Fig. 5D; Supplementary Table 1).

To examine whether CHD8 deficiency affected chromatin accessibility at CHD8 binding sites versus non-CHD8 binding sites, we overlapped ATAC peaks with CHIP peaks and found that CHD8 deficiency either increased or decreased chromatin accessibility at CHD8 binding sites (Fig. 5E; Supplementary Fig. 5G). There were 45 ATAC up and 31 ATAC down peaks at CHD8 binding sites upon CHD8 loss (Fig. 5E). Meanwhile, we found 946 ATAC up and 314 ATAC down peaks at non-CHD8 binding sites in the absence of CHD8 (Fig. 5F; Supplementary Fig. 5H). Our finding of ATAC up or ATAC down at CHD8 binding sites in Chd8^−/−^ Tregs suggests that chromatin closing or opening at certain genomic regions of Tregs is regulated by CHD8 direct binding. However, the finding of ATAC up or ATAC down at non-CHD8 binding sites in Chd8^−/−^ Tregs suggests that chromatin closing or opening at certain genomic regions of Tregs is regulated by CHD8 but not through CHD8 direct binding.

### Identification of CHD8 target genes whose expression is regulated by CHD8 direct binding-mediated chromatin remodeling

**Fig. 6:**
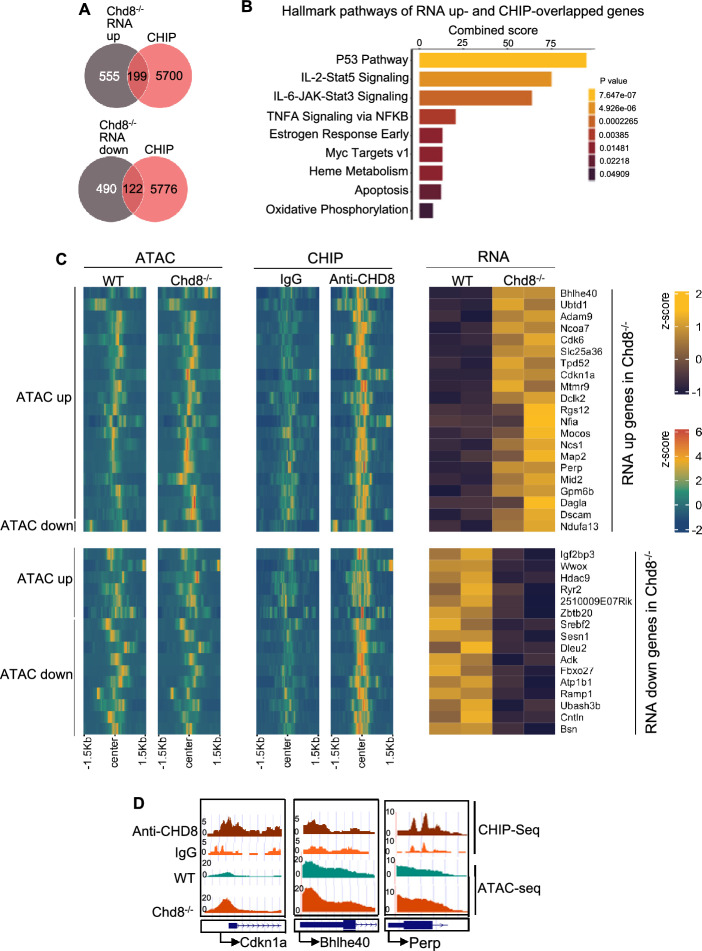
Identification of CHD8 target genes whose expression is regulated by CHD8 direct binding-mediated chromatin remodeling. **A** Overlapping between genes bound by CHD8 and genes upregulated or downregulated in Chd8^−/−^ cells identified through RNA-seq. RNA-seq Cutoff: Log2FC = 0.7. **B** Enriched pathways of CHD8 direct targets revealed by overlapping CHIP-seq data with RNA-seq data. Gene set enrichment was carried out by using Enrichr. **C** Heatmaps of RNA-seq, CHIP-seq, and ATAC-seq of CHD8 direct targets that may contribute to Chd8^−/−^ Treg phenotypes. **D** ATAC-seq and anti-CHD8 CHIP-seq tracks of Cdkn1a, Bhlhe40, and Perp in WT and/or Chd8^−/−^ Tregs

Next, we wished to identify CHD8 direct targets in Tregs. To this end, we overlapped CHD8 binding genes revealed by CHIP-seq with upregulated or downregulated genes in Chd8^−/−^ cells revealed by RNA-seq. We found that 199 genes (Supplementary Table 2) bound by CHD8 were upregulated and 122 genes (Supplementary Table 3) bound by CHD8 were downregulated upon CHD8 loss (Fig. 6A), suggesting that 321 genes are CHD8 direct targets, among which the expression of 199 genes is inhibited by CHD8 binding, and the expression of 122 genes is induced by CHD8 binding. Hallmark pathway analysis of the CHD8 direct targets revealed that a number of pathways were upregulated upon CHD8 deficiency. These pathways include p53, IL-2-Stat5, IL-6-JAK-Stat3, TNFA signaling via NFKB, Myc targets V1, apoptosis, and oxidative phosphorylation (Fig. 6B).

We then analyzed chromatin accessibility of the CHD8 direct targets by overlapping the upregulated or downregulated targets with genes bearing ATAC up or ATAC down in Chd8^−/−^ cells. We found that (1) 20 upregulated targets (Adam9, Bhlhe40, Cdk6, Cdkn1a, Dagla, Dclk2, Dscam, Gpm6b, Map2, Mid2, Mocos, Mtmr9, Ncoa7, Ncs1, Nfia, Perp, Rgs12, Slc25a36, Tpd52, and Ubtd1) showed ATAC up (Fig. 6C), suggesting that expression of those 20 genes is inhibited by CHD8 direct binding that closes the gene loci; (2) 1 upregulated target (Ndufa13) showed ATAC down (Fig. 6C), suggesting that expression of that gene is inhibited by CHD8 direct binding that opens the gene loci; (3) 6 downregulated targets (2510009E07Rik, Hdac9, Igf2bp3, Ryr2, Wwox, and Zbtb20) showed ATAC up (Fig. 6C), suggesting that expression of those 6 genes is promoted by CHD8 direct binding that closes the gene loci; and (4) 10 downregulated targets (Adk, Atp1b1, Bsn, Cntln, Dleu2, Fbxo27, Ramp1,Sesn1, Srebf2, and Ubash3b) showed ATAC down (Fig. 6C), suggesting that expression of those 10 genes is promoted by CHD8 direct binding that opens the gene loci. Collectively, our data indicate that expression of the above-mentioned genes is regulated by CHD8 binding-mediated chromatin remodeling, which may contribute to CHD8-mediated Treg phenotypes. To elaborate on this, CHD8-regulated expression of Cdkn1a, Bhlhe40 (a target of mTORC1, IL-2-Stat5, hypoxia, and TNFA signaling via NFKB), and Perp (a p53 target) appears to depend on CHD8 binding-mediated chromatin remodeling, as upregulation of Cdkn1a, Bhlhe40, and Perp in Chd8^−/−^ cells (Fig. 4D, E) was associated with increased chromatin accessibility at CHD8 binding sites (Fig. 6D).

### Treg-specific and Treg-nonspecific regulation of chromatin accessibility and gene expression by CHD8

Lastly, we examined whether CHD8 binding to DNA and its regulation of chromatin accessibility and gene expression in Tregs are specific to Tregs. We overlapped our CHIP-seq, ATAC-seq, and RNA-seq data with that from hematopoietic stem and progenitor cells (HSPCs) [9]. We found that among 6370 CHD8 binding genes in Tregs and 2261 CHD8 binding genes in HSPCs, 1069 CHD8 binding genes were shared by Tregs and HSPCs (Supplementary Table 4), whereas 5301 CHD8 binding genes in Tregs were specific to Tregs (Supplementary Table 5) and 1192 CHD8 binding genes in HSPCs were specific to HSPCs (Supplementary Table 6; Supplementary Fig. 6A). Among 854 ATAC up genes in Chd8^−/−^ Tregs and 1409 ATAC up genes in Chd8^−/−^ HSPCs, 134 ATAC up genes were shared by Tregs and HSPCs (Supplementary Table 7), whereas 720 ATAC up genes in Tregs were specific to Tregs (Supplementary Table 8), and 1275 ATAC up genes in HSPCs were specific to HSPCs (Supplementary Table 9; Supplementary Fig. 6B). Among 307 ATAC down genes in Chd8^−/−^ Tregs and 714 ATAC down genes in Chd8^−/−^ HSPCs, 24 ATAC down genes were shared by Tregs and HSPCs (Supplementary Table 10), whereas 283 ATAC down genes in Tregs were specific to Tregs (Supplementary Table 11), and 690 ATAC down genes in HSPCs were specific to HSPCs (Supplementary Table 12; Supplementary Fig. 6B). Among 218 RNA up genes in Chd8^−/−^ Tregs and 455 RNA up genes in Chd8^−/−^ HSPCs, 25 RNA up genes were shared by Tregs and HSPCs (Supplementary Table 13), whereas 193 RNA up genes in Tregs were specific to Tregs (Supplementary Table 14), and 430 RNA up genes in HSPCs were specific to HSPCs (Supplementary Table 15; Supplementary Fig. 6C). Among 167 RNA down genes in Chd8^−/−^ Tregs and 989 RNA down genes in Chd8^−/−^ HSPCs, 18 RNA down genes were shared by Tregs and HSPCs (Supplementary Table 16), whereas 149 RNA down genes in Tregs were specific to Tregs (Supplementary Table 17), and 971 RNA down genes in HSPCs were specific to HSPCs (Supplementary Table 18; Supplementary Fig. 6C). We also compared Tregs and HSPCs for hallmark pathway enrichment of CHD8 binding genes and genes with altered ATAC peaks or RNA expression in Chd8^−/−^ cells. While CHD8 binding genes in Tregs were enriched in PI3K-Akt-mTOR signaling, G2M checkpoint, E2F targets, and Myc targets (Fig. 5D), those in HSPCs were enriched in mTORC1 signing and E2F targets only (Supplementary Fig. 6D). Genes with altered ATAC peaks in Chd8^−/−^ Tregs showed enrichment in hypoxia and TNFA signaling via NFKB (Fig. 5B; Supplementary Fig. 5B), however, those in Chd8^−/−^ HSPCs showed enrichment in inflammatory response (Supplementary Fig. 6E). In addition, genes with altered RNA expression in Chd8^−/−^ Tregs were enriched in numerous pathways, including but not limited to Myc targets, E2F targets, mTORC1, p53, glycolysis, and oxidative phosphorylation (Fig. 4B and C). In contrast, genes with altered RNA expression in Chd8^−/−^ HSPCs were only enriched in Myc targets, oxidative phosphorylation, epithelial mesenchymal transition, and the p53 pathway (Supplementary Fig. 6F). Altogether, these results suggest that CHD8 plays a Treg-specific as well as universal role in Tregs.

## Discussion

In this study, we show that CHD8 deficiency in Tregs causes increased CD4^+^ and CD8^+^ effector T cells, leading to early, fatal inflammation in the skin and lung. Loss of CHD8 renders Tregs to become plastic, which may result in Treg reprogramming to CD4^+^ effector T cells. Plastic Tregs are generally thought to have dampened function in suppressing CD4^+^ and CD8^+^ effector T cells [4, 5]. In support, CHD8-deficient Tregs are incapable of competing with CHD8-proficient Tregs. Thus, it is conceivable that increased CD4^+^ effector T cells in CHD8-deficient mice are attributable to either Treg conversion or impaired Treg function or both, whereas increased CD8^+^ effector T cells are attributable to impaired Treg function.

Mechanistically, RNA-seq has found that CHD8 deficiency upregulates mTORC1 signaling and glycolysis. Given that mTOR signaling and its mediated glycolysis are associated with Treg plasticity [50, 51], CHD8 deficiency may induce Treg plasticity through upregulation of mTOR-glycolysis pathway. In support of this notion, mTOR target genes Cdkn1a and Bhlhe40 are CHD8 direct targets whose upregulation in the absence of CHD8 appears to be mediated by increased chromatin accessibility at CHD8 binding site of the gene loci. In addition, Chd8^−/−^ Treg plasticity may be attributed to upregulation of a number of inflammatory pathways revealed by hallmark pathway analysis of RNA-seq, ATAC-seq, and/or CHD8 direct targets, notably IL-2-Stat5 signaling, which is important for IFN-γ expression and IL-6-JAK-Stat3 pathway, which is essential for IL-17 expression.

CHD8 is well-known to regulate p53 and Wnt pathways. Our RNA-seq reveals that CHD8 deficiency in Tregs upregulates p53 pathway; however, it does not affect Wnt pathway (data not shown), suggesting that CHD8 modulates p53 but not Wnt-β-catenin signaling in Tregs, reminiscent of that in HSPCs [9]. p53 signaling promotes cell apoptosis and cell cycle arrest. Consistent with enhanced p53 signaling (e.g., Cdkn1a and Perp) in CHD8-deficient Tregs, CHD8 deficiency increases apoptosis of Tregs. Unexpectedly, CHD8 deletion accelerates Treg cell cycle progression/proliferation. Noting that Cdk6 promotes cell cycle progression and is a CHD8 direct target whose expression presumably depends on CHD8 binding-mediated chromatin remodeling, the increased Treg cell cycle progression may be mediated by upregulated Cdk6 and reflect a compensatory phenomenon of the increased cell apoptosis. Of note, p53 gene itself is not affected by CHD8 deficiency in both Tregs (data not shown) and HSPCs [9]. However, CHD8 deletion stabilizes p53 protein in HSPCs. Thus, it remains to be seen whether CHD8 deficiency increases p53 stability in Tregs.

It has been indicated that CHD8 regulates chromatin remodeling/accessibility in a cell type-specific manner. For example, CHD8 promotes chromatin opening in oligodendrocyte progenitors [21] but chromatin closing/compaction in HSPCs [9]. Our study reveals that CHD8 plays a binary role in chromatin remodeling in Tregs, as its deletion results in both ATAC up and ATAC down. How CHD8 regulates chromatin remodeling is not well-understood. We suggest that in Tregs, CHD8 regulates chromatin remodeling either dependent or independent of direct binding.

Taken together, by characterizing mouse models of Treg-specific deletion of autism-associated CHD8, we demonstrated that CHD8 plays an important role in maintaining Treg fitness, by manipulating genetic and epigenetic programs, to control autoimmunity. Of note, patients with autism have increased inflammatory cytokines IL-21, IL-22, IFN-γ, and IL-4 from T cells [16, 17], decreased antiinflammatory cytokines TGFβ and IL-10, two functional cytokines produced by Tregs [16–18], activated T cells, and/or autoimmunity [19]. Our findings suggest that the neuroinflammatory processes in patients with autism may be attributed to CHD8 deficiency in Tregs.

## Supplementary Information

Additional File 1: Fig. 1. Verification of deletion of Chd8 in Tregs. (A) Schema of mouse crossing to generate Chd8-/- mice. (B) Splenic Tregs (CD4+CD25+) were isolated from both genotypes of mice with a CD4+CD25+ regulatory T cell isolation kit. Cell purity was confirmed by FACS (B). The cells were used for PCR genotyping with primers for WT, Flox and KO allele of Chd8 or Foxp3-Cre (C). Total RNA was extracted from Tregs for qPCR to confirm deletion of Chd8. The mRNA levels of Chd8 are shown (arbitrary unit). The data are normalized to an 18S reference and expressed as mean plus SD of triplicates representative of two separated experiments (D). **P < 0.01 vs. WT control group. Fig. 2. CHD8 deficiency increases CD8+ T cells. Splenocytes were analyzed for CD4+ and CD8+ T cells by FACS. Representative dot plots are shown. The proportions and absolute numbers of CD4+ and CD8+ cells are summarized in bar graphs (mean plus SD) (WT: 6 males + 6 females; Chd8-/-: 4 males + 6 females). *P < 0.05, **P < 0.01 vs. WT control group. Fig. 3. CHD8 deficiency does not affect overt thymocyte and thymic Treg development. Thymocytes were analyzed for CD4-CD8- (DN), CD4+CD8+ (DP), CD4+CD8- (SP4), CD4-CD8+ (SP8) (A), and CD4+Foxp3+ (B) cells by FACS. Representative dot plots are shown. The proportions and absolute numbers of the cells are summarized in bar graphs (mean plus SD) (WT: 3 males; Chd8-/-: 3 males). *P < 0.05 vs. WT control group. Fig. 4. Verification of gene expression changes in p53 and mTOR pathways by quantitative real-time RT-PCR. The expression changes of the indicated genes in p53 (A) and mTOR (B) pathways in Chd8-/- Tregs revealed by RNA-seq as shown in Fig 4E were verified by qPCR. The data are normalized to an 18S reference and expressed as mean + SD of triplicates. *P < 0.05, **P < 0.01 vs. WT control group. Fig. 5. Profiles and enrichment plots of ATAC-seq and CHIP-seq A PCA of ATAC-seq. B Fragment size distribution of ATAC-seq. C Profile of ATAC-seq peaks in Chd8-/- (vs WT) Tregs. D GSEA enrichment plots of ATAC up pathways in Chd8-/- Tregs. E PCA of CHIP-seq. F Pie plot of distribution of CHD8 binding peaks in the genome of Tregs. The proportion of peaks in an indicated genomic region was calculated by dividing the number of peaks in that region by the total number of peaks in the genome. The proportion of an indicated genomic region size was determined by dividing the region’s size by the total genome size. The proportion of peaks was then normalized to the proportion of genomic region size. Normalized proportion of peaks is shown. G Profile of ATAC up and ATAC down peaks at CHD8 binding sites in Chd8-/- (vs WT) Tregs. H Profile of ATAC up and ATAC down peaks at non-CHD8 binding sites in Chd8-/- (vs WT) Tregs. Fig. 6. Treg-specific and -nonspecific patterns of CHD8 in DNA binding and regulation of chromatin accessibility and gene expression. A Overlapping of CHD8 binding genes between Tregs and HSPCs. B Overlapping of ATAC up or ATAC down genes between Chd8-/- Tregs and Chd8-/- HSPCs. Cutoff: Log2FC = 0.4. C Overlapping of RNA up or RNA down genes between Chd8-/- Tregs and Chd8-/- HSPCs. Cutoff: Log2FC = 1.0. D Hallmark pathways of CHD8 binding genes in HSPCs. E Hallmark pathways of genes with altered ATAC peaks in Chd8-/- versus WT HSPCs. F Hallmark pathways of genes with altered RNA expression in Chd8-/- versus WT HSPCs.

## Data Availability

All data relevant to the study are included in the article, uploaded as online supplemental information, or deposited in the National Center for Biotechnology Information GEO (accession no. GSE279810).

## Abbreviations

ATAC-seq: Assay for transposase-accessible chromatin sequencing
BrdU: Bromodeoxyuridine
CHD8: Chromodomain helicase DNA-binding protein 8
CHIP-seq: Chromatin immunoprecipitation sequencing
E2F: Early region 2 binding factor
Flox: Floxed, flanked by loxP
Foxp3: Forkhead box P3
GITR: Glucocorticoid-induced TNFR-related protein
H&E: Hematoxylin and eosin
HSPC: Hematopoietic stem and progenitor cell
ICOS: Inducible T cell costimulatory
IL-4: Interleukin-4
KO: Knockout
MFI: Mean fluorescence intensity
mTORC1: Mammalian target of rapamycin complex 1
NFKB: Nuclear factor kappa-light-chain-enhancer of activated B cells
PBS: Phosphate buffer saline
PD-1: Programmed death 1
PI3K/Akt/mTOR: Phosphatidylinositol-3 kinase-protein kinase B-mammalian target of rapamycin
qPCR: Quantitative real-time polymerase chain reaction
RNA-seq: RNA sequencing
ROS: Reactive oxygen species
Th: T helper
TNFA: Tumor necrosis factor alpha
Tregs: Regulatory T cells
WT: Wild type
YFP: Yellow fluorescent protein
