# Supplementary figures and images for: Genetic and epigenetic regulation of Treg cell fitness by autism-related chromatin remodeler CHD8
Source: Cell Mol Biol Lett. 2025 Mar 28;30:36. doi: 10.1186/s11658-025-00711-z (PMC11954365; doi:10.1186/s11658-025-00711-z)

Fig. S1

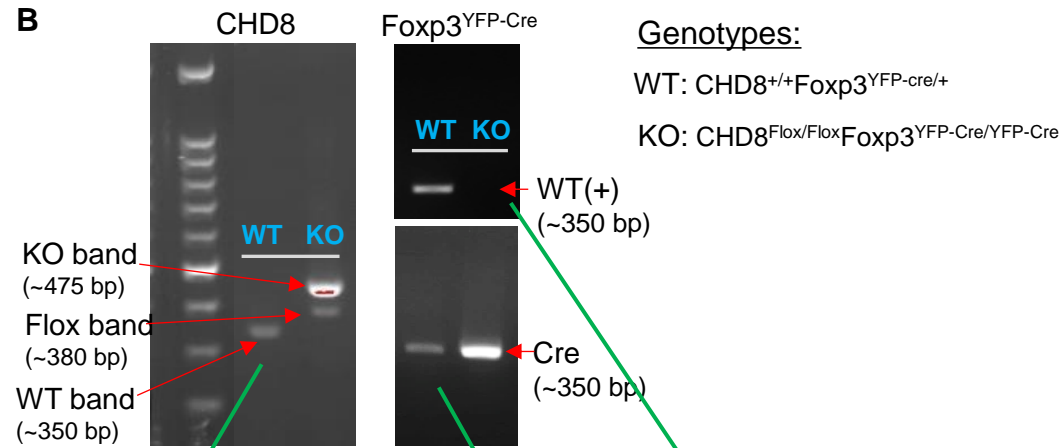

Uncropped gels

CHD8-flox PCR

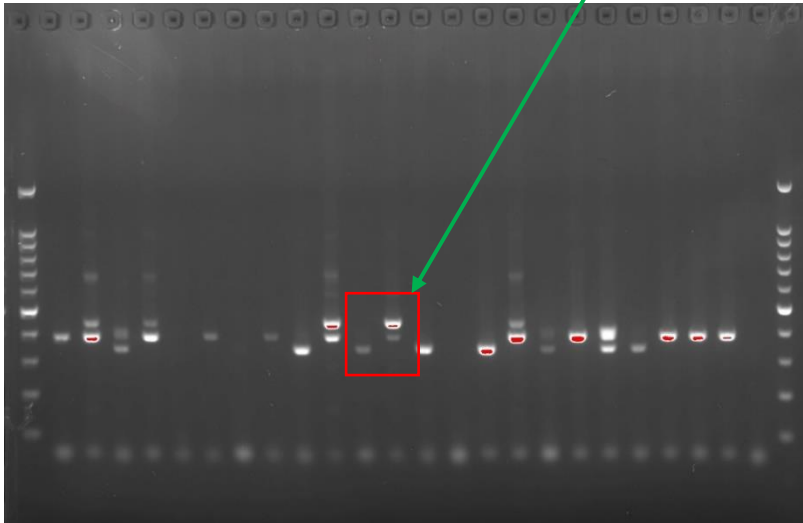

Foxp3-Cre PCR

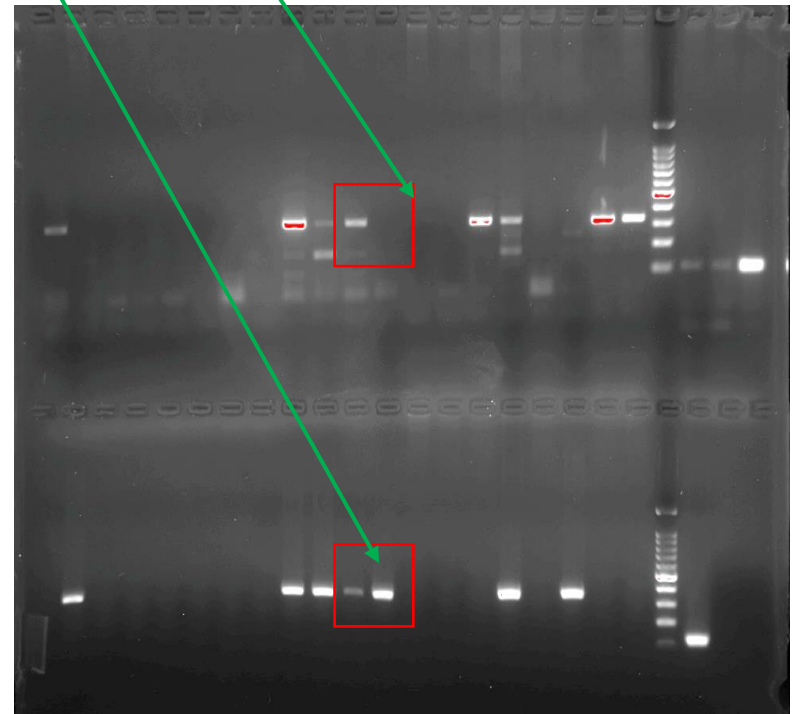

Supplement: Supplementary file 1 — Additional File 1: Fig. 1. Verification of deletion of Chd8 in Tregs. (A) Schema of mouse crossing to generate Chd8-/- mice. (B) Splenic Tregs (CD4+CD25+) were isolated from both genotypes of mice with a CD4+CD25+ regulatory T cell isolation kit. Cell purity was confirmed by FACS (B). The cells were used for PCR genotyping with primers for WT, Flox and KO allele of Chd8 or Foxp3-Cre (C). Total RNA was extracted from Tregs for qPCR to confirm deletion of Chd8. The mRNA levels of Chd8 are shown (arbitrary unit). The data are normalized to an 18S reference and expressed as mean plus SD of triplicates representative of two separated experiments (D). **P < 0.01 vs. WT control group. Fig. 2. CHD8 deficiency increases CD8+ T cells. Splenocytes were analyzed for CD4+ and CD8+ T cells by FACS. Representative dot plots are shown. The proportions and absolute numbers of CD4+ and CD8+ cells are summarized in bar graphs (mean plus SD) (WT: 6 males + 6 females; Chd8-/-: 4 males + 6 females). *P < 0.05, **P < 0.01 vs. WT control group. Fig. 3. CHD8 deficiency does not affect overt thymocyte and thymic Treg development. Thymocytes were analyzed for CD4-CD8- (DN), CD4+CD8+ (DP), CD4+CD8- (SP4), CD4-CD8+ (SP8) (A), and CD4+Foxp3+ (B) cells by FACS. Representative dot plots are shown. The proportions and absolute numbers of the cells are summarized in bar graphs (mean plus SD) (WT: 3 males; Chd8-/-: 3 males). *P < 0.05 vs. WT control group. Fig. 4. Verification of gene expression changes in p53 and mTOR pathways by quantitative real-time RT-PCR. The expression changes of the indicated genes in p53 (A) and mTOR (B) pathways in Chd8-/- Tregs revealed by RNA-seq as shown in Fig 4E were verified by qPCR. The data are normalized to an 18S reference and expressed as mean + SD of triplicates. *P < 0.05, **P < 0.01 vs. WT control group. Fig. 5. Profiles and enrichment plots of ATAC-seq and CHIP-seq A PCA of ATAC-seq. B Fragment size distribution of ATAC-seq. C Profile of ATAC-seq peaks in Chd [file 11658_2025_711_MOESM1_ESM.zip › supplement/Fig S1B uncropped gels.pdf]

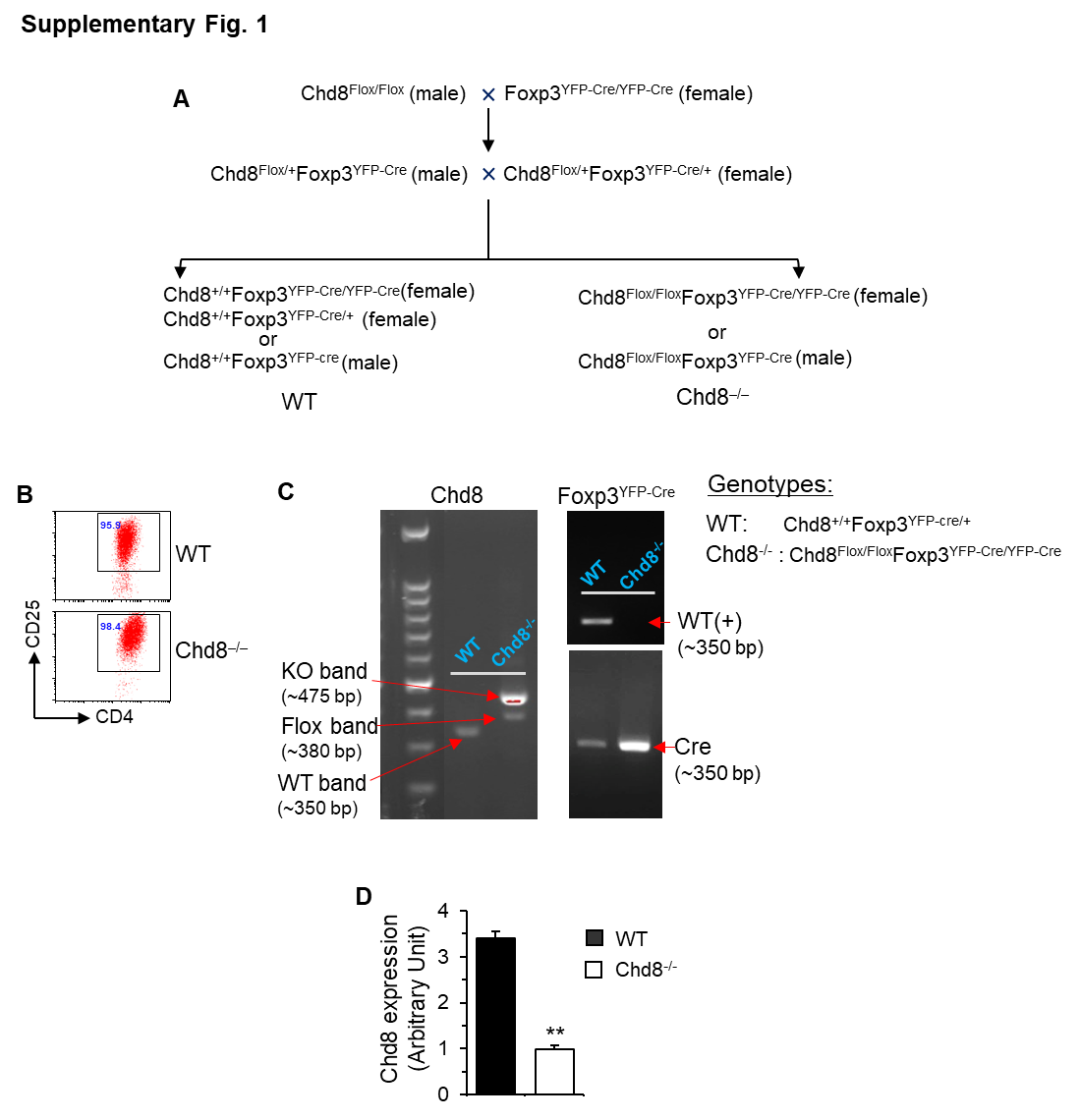


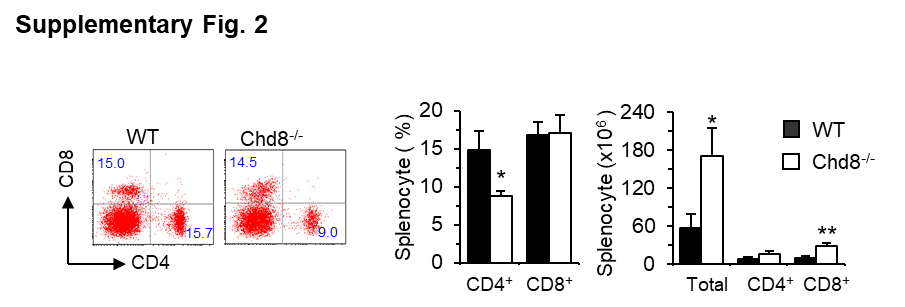


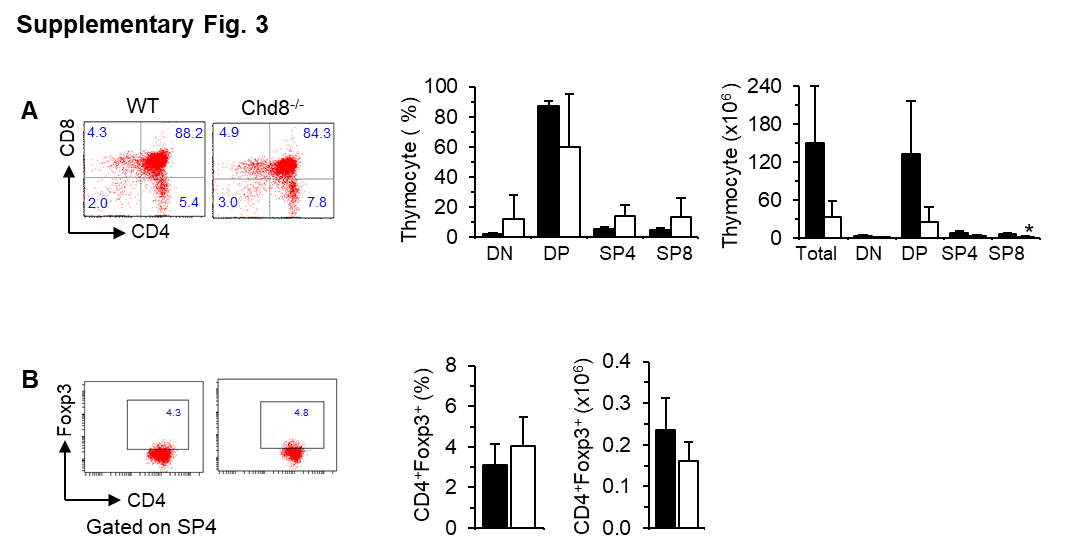


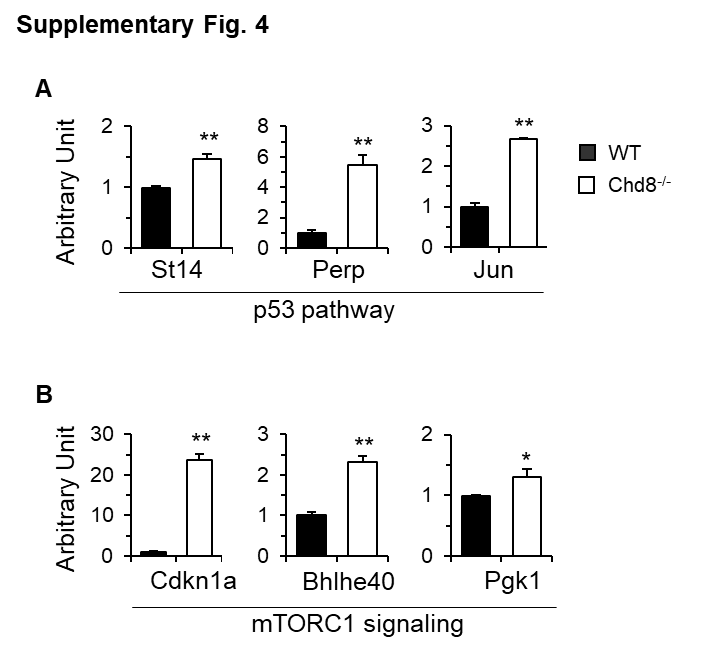


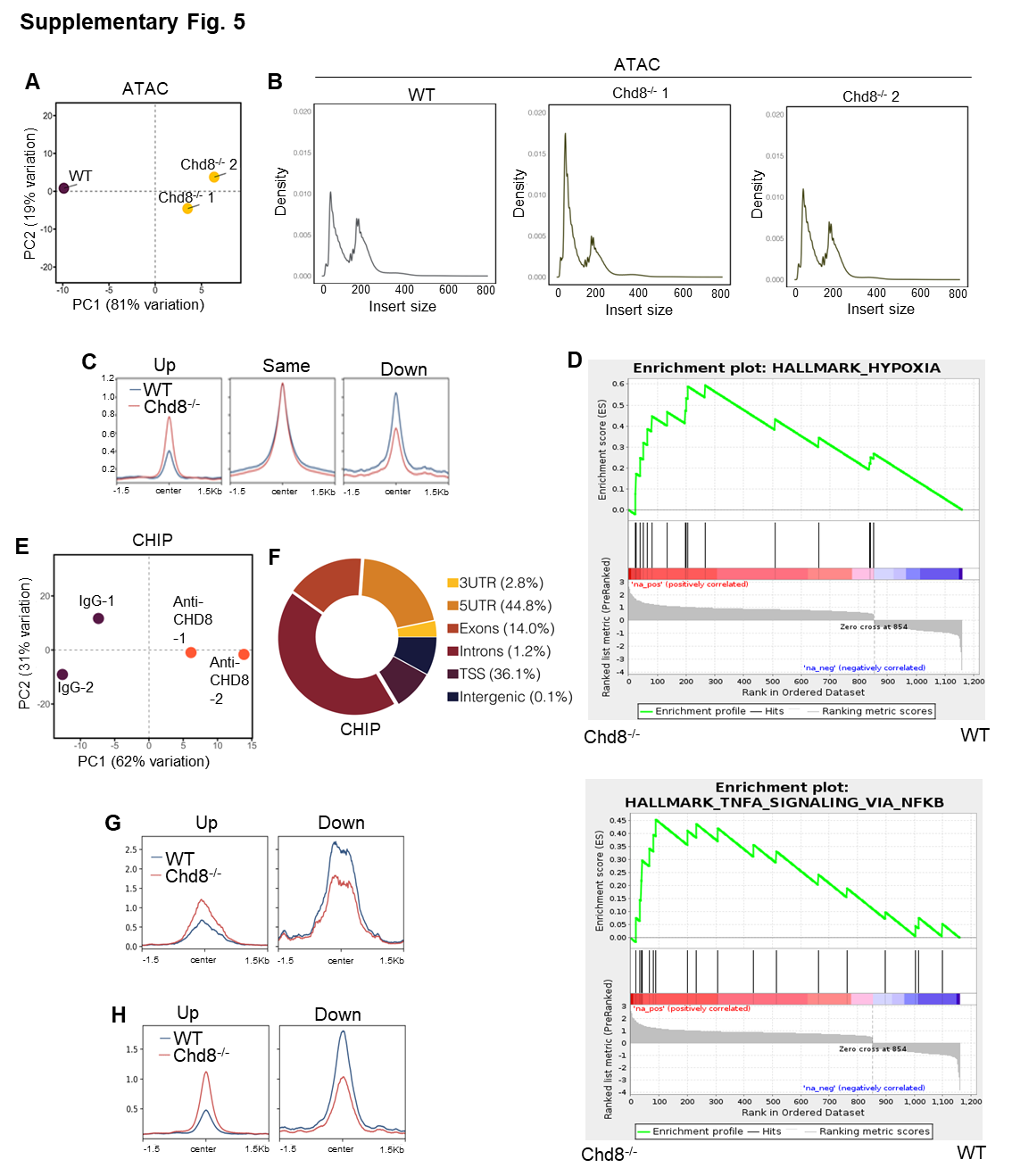


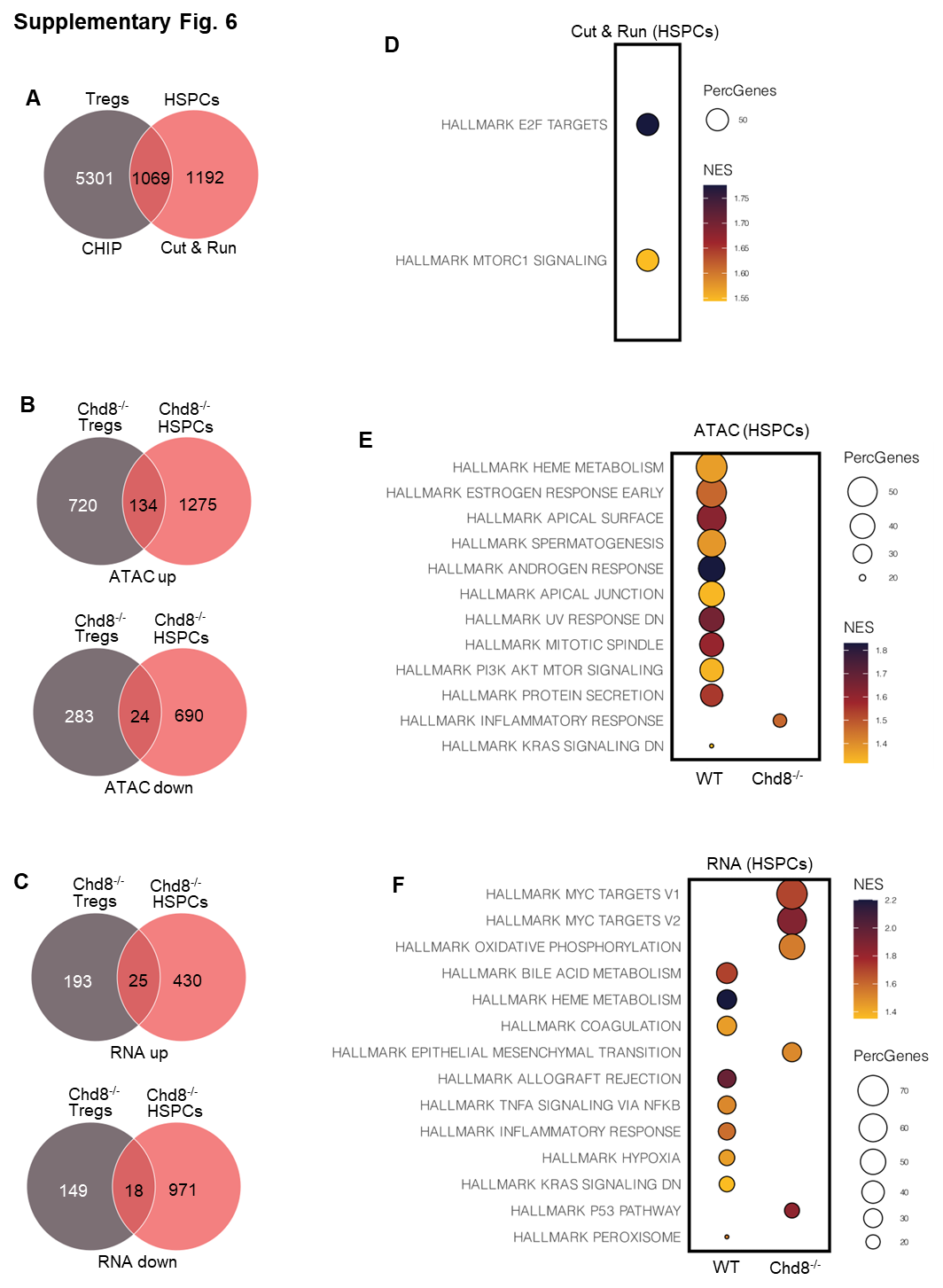

Supplement: Supplementary file 1 — Additional File 1: Fig. 1. Verification of deletion of Chd8 in Tregs. (A) Schema of mouse crossing to generate Chd8-/- mice. (B) Splenic Tregs (CD4+CD25+) were isolated from both genotypes of mice with a CD4+CD25+ regulatory T cell isolation kit. Cell purity was confirmed by FACS (B). The cells were used for PCR genotyping with primers for WT, Flox and KO allele of Chd8 or Foxp3-Cre (C). Total RNA was extracted from Tregs for qPCR to confirm deletion of Chd8. The mRNA levels of Chd8 are shown (arbitrary unit). The data are normalized to an 18S reference and expressed as mean plus SD of triplicates representative of two separated experiments (D). **P < 0.01 vs. WT control group. Fig. 2. CHD8 deficiency increases CD8+ T cells. Splenocytes were analyzed for CD4+ and CD8+ T cells by FACS. Representative dot plots are shown. The proportions and absolute numbers of CD4+ and CD8+ cells are summarized in bar graphs (mean plus SD) (WT: 6 males + 6 females; Chd8-/-: 4 males + 6 females). *P < 0.05, **P < 0.01 vs. WT control group. Fig. 3. CHD8 deficiency does not affect overt thymocyte and thymic Treg development. Thymocytes were analyzed for CD4-CD8- (DN), CD4+CD8+ (DP), CD4+CD8- (SP4), CD4-CD8+ (SP8) (A), and CD4+Foxp3+ (B) cells by FACS. Representative dot plots are shown. The proportions and absolute numbers of the cells are summarized in bar graphs (mean plus SD) (WT: 3 males; Chd8-/-: 3 males). *P < 0.05 vs. WT control group. Fig. 4. Verification of gene expression changes in p53 and mTOR pathways by quantitative real-time RT-PCR. The expression changes of the indicated genes in p53 (A) and mTOR (B) pathways in Chd8-/- Tregs revealed by RNA-seq as shown in Fig 4E were verified by qPCR. The data are normalized to an 18S reference and expressed as mean + SD of triplicates. *P < 0.05, **P < 0.01 vs. WT control group. Fig. 5. Profiles and enrichment plots of ATAC-seq and CHIP-seq A PCA of ATAC-seq. B Fragment size distribution of ATAC-seq. C Profile of ATAC-seq peaks in Chd [file 11658_2025_711_MOESM1_ESM.zip › supplement/supplement.docx]
